# Non-medical factors in prehospital resuscitation decision-making: a mixed-methods systematic review

**DOI:** 10.1186/s13049-022-01004-6

**Published:** 2022-03-28

**Authors:** Louise Milling, Jeannett Kjær, Lars Grassmé Binderup, Caroline Schaffalitzky de Muckadell, Ulrik Havshøj, Helle Collatz Christensen, Erika Frischknecht Christensen, Annmarie Touborg Lassen, Søren Mikkelsen, Dorthe Nielsen

**Affiliations:** 1 Prehospital Research Unit, Department of Anaesthesiology and Intensive Care, Odense University Hospital, Kildemosevej 15, 5000 Odense C, Denmark; 2 Department of Regional Health Research, University of Southern Denmark, Odense, Denmark; 3 Philosophy, Department for the Study of Culture, University of Southern Denmark, Odense, Denmark; 4 The Danish Clinical Quality Program, National Clinical Registries, Copenhagen, Denmark; 5 Centre for Prehospital and Emergency Research, Aalborg University Hospital, Aalborg University, Aalborg, Denmark; 6 Emergency Medical Services, Region North Denmark, Aalborg, Denmark; 7 Emergency Medicine Research Unit, Odense University Hospital, Odense, Denmark; 8 Department of Infectious Diseases, Sub-Department of Immigrant Medicine, Odense University Hospital, Odense, Denmark; 9 Department of Geriatric Medicine, Odense University Hospital, Odense, Denmark

**Keywords:** Ethics, Decision-making, Out-of-hospital cardiac arrest

## Abstract

**Aim:**

This systematic review explored how non-medical factors influence the prehospital resuscitation providers’ decisions whether or not to resuscitate adult patients with cardiac arrest.

**Methods:**

We conducted a mixed-methods systematic review with a narrative synthesis and searched for original quantitative, qualitative, and mixed-methods studies on non-medical factors influencing resuscitation of out-of-hospital cardiac arrest. Mixed-method reviews combine qualitative, quantitative, and mixed-method studies to answer complex multidisciplinary questions. Our inclusion criteria were peer-reviewed empirical-based studies concerning decision-making in prehospital resuscitation of adults > 18 years combined with non-medical factors. We excluded commentaries, case reports, editorials, and systematic reviews. After screening and full-text review, we undertook a sequential exploratory synthesis of the included studies, where qualitative data were synthesised first followed by a synthesis of the quantitative findings.

**Results:**

We screened 15,693 studies, reviewed 163 full-text studies, and included 27 papers (12 qualitative, two mixed-method, and 13 quantitative papers). We identified five main themes and 13 subthemes related to decision-making in prehospital resuscitation. Especially the patient’s characteristics and the ethical aspects were included in decisions concerning resuscitation. The wishes and emotions of bystanders further influenced the decision-making. The prehospital resuscitation providers’ characteristics, experiences, emotions, values, and team interactions affected decision-making, as did external factors such as the emergency medical service system and the work environment, the legislation, and the cardiac arrest setting. Lastly, prehospital resuscitation providers’ had to navigate conflicts between jurisdiction and guidelines, and conflicting values and interests.

**Conclusions:**

Our findings underline the complexity in prehospital resuscitation decision-making and highlight the need for further research on non-medical factors in out-of-hospital cardiac arrest.

**Supplementary Information:**

The online version contains supplementary material available at 10.1186/s13049-022-01004-6.

## Background

Out-of-hospital cardiac arrest is associated with poor survival rates [1]. The initial professional treatment of out-of-hospital cardiac arrest patients requires the prehospital resuscitation providers (providers) to decide whether to withhold, initiate, continue, or terminate the prehospital cardiopulmonary resuscitation. This medical decision-making is a deliberative process that combines conscious and unconscious influences derived from medical evidence, personal and medical beliefs, and, where possible, knowledge of patient preferences [2]. Clinical guidelines based on medical factors, such as comorbidities or initial cardiac rhythm assist decision-making in prehospital resuscitation in some countries, while other countries rely on individual judgement [3]. Even though resuscitation decision-making is based on medical evidence, non-medical factors can influence decision-making, even where guidelines and clinical rules are applied, which may in turn influence patient survival [2, 4].

Anderson et al. described the factors influencing the treatment of out-of-hospital cardiac arrest and identified five themes: the arrest event, the patient characteristics, the resuscitation scene, the perspective of the resuscitation provider, and medico-legal concerns [2]. The study explored both medical and non-medical factors and found that non-medical factors may change the course of action especially if the medical factors point in diverging directions [2]. As the field of resuscitation evolves, the context in which healthcare professionals make decisions changes. Recent studies describe new aspects, including non-medical factors, in decision-making in prehospital resuscitation [5–9]. We aimed to review original studies on non-medical factors that pre-hospital care providers describe as important for decision-making in adult resuscitation..

## Methods

We conducted a mixed-methods systematic review with a narrative synthesis and reported this according to the Cochrane Qualitative Research Methods Group guidelines [10] and the PRISMA guidelines for reporting of systematic reviews [11]. As PRISMA is not designed for mixed-methods systematic reviews, we applied the fields relevant to our study (See Additional file 1). A systematic mixed-method review allows for the inclusion of multiple methodologies to combine the strengths of quantitative and qualitative methods but also makes it possible to compensate for the limitations in each method [12]. Our protocol was published on PROSPERO on March 17, 2021 (PROSPERO registration number CRD42021237078).

### Selection criteria and search strategy

We searched for peer-reviewed quantitative, qualitative, and mixed-methods studies containing empirical data on non-medical factors in prehospital resuscitation of out-of-hospital cardiac arrest and their influence on the providers’ decision-making. Non-medical factors are medically extraneous factors, not necessarily based on symptoms or tests, and therefore can be difficult to define precisely [13, 14]. We used the existing literature on non-clinical and non-medical factors to outline the definition of non-medical factors [15–17]. This literature does not necessarily originate from the emergency medical setting and the non-medical factors had to be adapted to the prehospital resuscitation decision-making. See Table 1 for examples. We included studies concerning decision-making in prehospital resuscitation of adults > 18 years and non-medical factors as described by the resuscitation providers employed in EMS systems. We only included mixed populations of in-hospital healthcare personnel and prehospital providers if we could determine that the majority of the study population were EMS workers. Mixed populations of paediatric patients and adult patients were included if the majority of the patients were adults. We contacted the authors of two studies with a mixed population for additional information on the proportion of prehospital participants. We excluded studies concerning in-hospital decision-making, paediatric resuscitation, post-resuscitation care, and register-based studies where decision-making was not described by the providers.

**Table 1:** Examples of non-medical factors

*Patient-related factors* Patient’s socioeconomic status Patient’s race Patient’s age, gender, and other personal characteristics Patient’s wishes and preferences (e.g. do not attempt cardiopulmonary resuscitation orders) Relative’s opinions and attitudes Patient’s religion and faith Patient’s quality of life *Prehospital resuscitation provider-related factors* PRP’s characteristics, age, gender, culture, faith, and race PRP’s time constraints and work overload in a clinical situation PRP’s interaction with other resuscitation providers PRP’s perception of the outcome *Setting* Concerns of own safety Legal implications	

We conducted our search guided by the PRISMA Search Report Extension [18]. A research librarian assisted the search in the following electronic databases: PubMed, MEDLINE, EMBASE, CINDAHL, Cochrane Library, and PsycINFO (See Additional files 2 and 3 for the full search strategy and the list of search terms). We performed the final search on June 11, 2021. To ensure data completion, we used Scopus to identify articles that cited the included papers and used the snowballing method to identify additional eligible studies from the reference lists of the included studies [19] and screened the reference lists of systematic reviews meeting our inclusion criteria for eligible studies. We used Covidence (Covidence systematic review software, Veritas Health Innovation, Melbourne, Australia. Available at www.covidence.org) for data management. Authors LM and JK conducted the title and abstract screening independently. Conflicts were resolved in consultation with author SM.

### Data extraction

Authors LM and JK independently extracted data on study title, journal title, methodology, and participants, setting characteristics (e.g., country of origin, type of emergency medical system, legislation concerning termination of resuscitation), phenomena of interest, and outcomes of relevance to the review questions. Qualitative data consisted of themes and subthemes including relevant illustrations. Quantitative data consisted of outcomes based on descriptive or inferential statistical tests. Disagreements were resolved through discussion with author DN.

### Assessment of methodological quality

Authors LM and JK critically appraised the included studies independently using the mixed methods appraisal tool (MMAT) [20]. The MMAT assesses both qualitative, quantitative, and mixed methods studies using different templates for each method. Every template contains five different criteria to be assessed, thus allowing one robust score to be used for multiple study types [20]. This enables appraisal scores ranging from 0% (no criteria met) to 100% (all five criteria met).

### Data synthesis

We undertook a sequential exploratory synthesis in which qualitative data are synthesised first followed by a synthesis of the quantitative findings to generalise and test the qualitative findings [12]. The findings are then incorporated into an overall synthesis and interpretation. Our qualitative synthesis consisted of a thematic content analysis inspired by Malterud [21]. The subsequent quantitative synthesis including a meta-analysis was not possible due to heterogeneity of interventions and outcome measures, and instead, we used a narrative approach. In the final synthesis, we incorporated the quantitative findings with the themes and subthemes identified during the thematic content analysis. See Additional file 4.

### Ethics

Due to the nature of the study, ethical approval was not required.

## Results

### Study selection and study characteristics

**Fig. 1:**
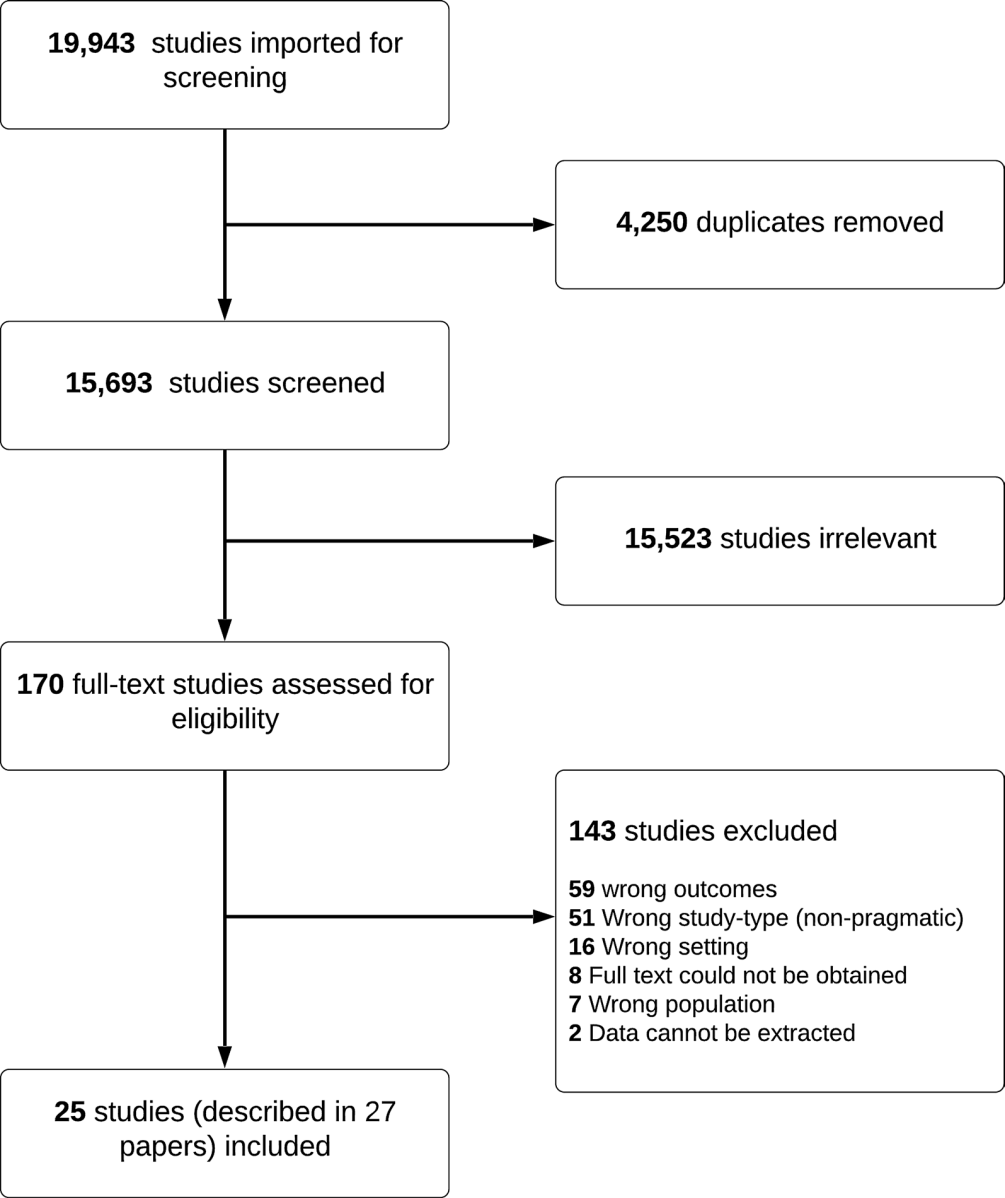
PRISMA flowchart of the inclusion process

We identified 19,943 papers through database searches and included 27 papers after screening. Of the 143 papers excluded after full-text review, 59 had study outcomes irrelevant to our study such as decision-making factors based on registry data and not data described by providers, 51 were opinion or discussion papers without data, 16 were from an in-hospital setting, seven contained in-hospital providers’ opinions on OHCA. In eight papers, a full-text article could not be obtained, and two papers were abstracts without full-texts available. See Fig. 1 for a flowchart of the study process. The included papers contained data from 25 unique studies. Of these papers, 12 were qualitative studies [5, 8, 22–31], two mixed-method studies [32, 33], and 13 quantitative studies [34–46]. The papers were published between 1993 and 2021. See Table 2 for the study characteristics. Of the included papers, 24 concerned prehospital providers only, while three papers [34, 35, 42] included both prehospital and in-hospital healthcare professionals (with a majority of prehospital providers). See Table 3 for additional study characteristics.

**Table 2:** Study characteristics and critical appraisal score

Study (references)	Country	Aim of the study	Participants	n	Age	Gender	Experience	Quality assessment score % (Median (range))
*Qualitative studies*								
Anderson et al. [8]	New Zealand	To identify the clinical, ethical, cognitive, and emotional challenges that emergency ambulance personnel experience when making decisions to commence, continue, withhold or terminate resuscitation	Ambulance personnel (first responder, EMT, paramedic, intensive care paramedic)	16	< 25–64 years	8 male 8 female	2–38 years Median: 12 years	100
Anderson et al. [8]	New Zealand	To explore ambulance personnel’s decisions to commence, continue, withhold or terminate resuscitation efforts for patients with OHCA	Ambulance personnel (first responder, EMT, paramedic, intensive care paramedic)	16	< 25–64 years	8 male 8 female	2–38 years Median: 12 years	100
Brandling et al. [23]	United Kingdom	To explore the influences on UK EMS provider decision-making when commencing and ceasing resuscitation attempts in OHCA	Paramedics	16	Median age: 40 years	10 male 6 female	Median: 15 years	80
Bremer et al. [24]	Sweden	To analyze EMS personnel’s experiences of caring for families when patients suffer cardiac arrest and sudden death	Specialist nurse (intensive care), paramedic (assistant nurse), prehospital emergency nurse, paramedic, specialist nurse (anesthesia)	10	26–62 years	6 male 4 female	< 1—> 20 years	100
Davey et al. [25]	New Zealand	To highlight and explore underlying values present within practice-based decisions that focus on ADs	EMT, intermediate life support, and intensive care paramedic	18	12 participants were aged > 30 years and four < 30 years	13 male 4 female 1 undisclosed	> 3—> 10 years	60
Karlsson et al. [26]	Sweden	To investigate Swedish specialist ambulance nurses’ experiences of ethical dilemmas associated with cardiac arrest situations in adult patients’ homes	Specialist ambulance nurses	9	33–61 years Mean: 45.5	4 male 5 female	5–17 years Mean: 11.5	100
Larsson et el. [27]	Sweden	To describe ambulance nurses’ experiences of nursing patients suffering cardiac arrest	Ambulance nurses	7	35–52 years	5 male 2 female	2–6 years Median: 11 years	100
Leemeyer et al. [5]	The Nether-lands	To identify factors that influence decision-making by prehospital EMS providers during resuscitation of patients with traumatic cardiac arrest	Ambulance nurses, HEMS nurses, and HEMS physicians	25	39–48 years Median age: 43	18 male 7 female	9–20 years Median: 12 years	100
Lord et al. [28]	Australia	To describe outcomes of the first phase of a larger research project exploring the interface between paramedics and patients who require palliative care	Paramedics	25	The majority were aged under 40 years	23 male 2 female	NA	80
Naess et al. [29]	Norway	To elucidate the criteria used by the paramedics in the Oslo EMS system when making decisions about CPR and whether these criteria tended to differ from the criteria used by the doctors on the physician manned ambulance and if they were affected by the length of experience	Paramedics, residents, and staff anesthesiologists	44	NA	41 male 3 female	1—> 20 years	0
Nordby et al. [30]	Norway	To understand how paramedics experience difficult ethical dilemmas regarding resuscitation of cancer patients	Paramedics	15	NA	NA	NA	100
Nurok et al. [31]	United States France	To analyze the role of social, technical, medical or surgical, heroic, and competence values in the course of pre-hospital emergency work	Prehospital Emergency Services	NA	NA	NA	NA	100
*Quantitative studies*								
Druwé et al. [35]	Austria, Belgium, France, Germany, Netherland, Republic of Ireland, UK, Czech Republic, Hungary, Poland, Romania, Serbia, Slovak Republic, Cyprus, Greece, Spain, Finland, Iceland, Norway, Sweden, Chile, Israel, Japan, United States	To determine the prevalence of clinician perception of inappropriate CPR regarding the last OHCA encountered in an adult 80 years or older and its relationship to patient outcome	Doctors, nurses, and EMTs/paramedics	611	NA	NA	NA	80
Druwé et al. [34]	Austria, Belgium, France, Germany, Netherland, Republic of Ireland, UK, Czech Republic, Hungary, Poland, Romania, Serbia, Slovak Republic, Cyprus, Greece, Spain, Finland, Iceland, Norway, Sweden, Chile, Israel, Japan, United States	To determine the prevalence of perception of inappropriate CPR of the last cardiac arrest encountered by clinicians working in emergency departments and out-of-hospital, factors associated with perception, and its relation to patient outcome	Doctors, nurses, and EMTs/paramedics	4018	NA	2409 male 1609 female	NA	80
Haidar et al. [36]	Lebanon	To examine the perspective of prehospital providers on resuscitation practices to inform and shape policy development related to resuscitation of OHCA victims in Lebanon	Prehospital providers (volunteers)	258	18- > 40 years	161 male 97 female	< 1—> 5 years	80
Hick et al. [37]	United States	To determine the factors that influence the transport of OHCA patients and to define problems with field termination of resuscitation efforts	Paramedics	259	NA	NA	NA	80
Johnson et al. [38]	United States	To examine occasions when EMTs do not initiate CPR according to their teaching or protocols. Furthermore, whether these situations troubled EMTs	EMTs	310	Mean age: 33.7 ± 8.2 years (SD)	235 male 75 female	Mean: 7.3 ± 7.2 years (SD)	100
Leibold et al. [39]	Germany	To detect whether or not religious and spiritual beliefs influence paramedics in their workday life concerning end-of-life decisions, and whether it is legally possible for them to act according to their conscience	Paramedics	429	Median age: 31 years	NA	Median: 8 years	80
Meyer et al. [40]	Germany	To introduce a new methodological approach towards initiation and termination of resuscitation efforts in prehospital situations	Emergency physicians	86	Mean age: 33.9	> 50% male	Mean: > 10 months	60
Mohr et al. [41]	Germany	To determine, by questioning emergency physicians, the time interval within which resuscitative efforts are usually terminated and the importance attached to the different factors concerning the decision to abandon CPR attempts	Emergency physicians	409	NA	NA	< 1—> 5 years	80
Navalpotro-Pascual et al. [42]	Spain	To explore the attitudes of the professionals that assist cardiopulmonary arrest in the face of these situations, and the factors that may influence them	Emergency physicians and nurses	1000 (593 OH)	Median age: 42 years	57% male 43% female	< 10—> 20 years	80
Sam et al. [43]	United States	To identify factors that influence the behavior of EMS professionals in seeking and honoring ADs. It specifically examined potential barriers affecting the implementation of ADs by EMS professionals	EMS professionals and volunteers	230	19–76 years Mean age: 35.2	70% male 30% female	NA	80
Sherbino et al. [44]	Canada	To estimate how frequently EMT-Ds are forced to deal with prehospital DNACPR orders, to assess their comfort in doing so, and to describe the prehospital care provided to patients with DNACPR orders in a system without a prehospital DNACPR policy (i.e., where resuscitation is mandatory)	Emergency physicians	221	NA	NA	1–30 years Mean: 14 years	60
Stone et al. [45]	United States	To ascertain paramedics' attitudes toward end-of-life situations and the frequency with which they encounter them, and to compare paramedics' preparation during training for a variety of end-of-life care skills	Paramedics	235	22–59 years Mean age: 39 years	94% male 6% female	< 2—> 20 years	60
Tataris et al. [46]	United States	To identify EMS providers’ perceived barriers to performing out-of-hospital TOR in a large urban EMS system	Firefighter/EMT-basic, firefighter/EMT-paramedic and single role paramedic	2309	NA	NA	Median: 16 years	100
*Mixed methods studies*								
de Graaf et al. [32]	The Netherlands	To determine differences between patients without ROSC to be transported vs. terminated on scene and explore medical and nonmedical factors that contribute to the decision-making of paramedics on scene	Paramedics	QUAL: 16	Median age: 49 years	10 male 6 female	> 1 years	100
Waldrop et al. [33]	United States	To explore prehospital providers’ perspectives on how legally binding documents (nonhospital DNACPR order/medical orders for life-sustaining treatment) informed end-of-life decision-making and care	Resuscitation providers	QUAN: 239 QUAL: 50	Mean age: 34.6 ± 11.8 (SD)	77% male 23% female	NA	20

n, number of participants; Experience, Years of experience in emergency medicine; OH, out-hospital; NA, not available; EMT, Emergency medical technician; EMT-D, EMTs with defibrillation skills; OHCA, out-of-hospital cardiac arrest; EMS, emergency medical service; TOR, termination of resuscitation; SD, standard deviation; CPR, cardiopulmonary resuscitation; DNACPR, do not attempt CPR HEMS, Helicopter Emergency Medical Service; AD, advance directives; ROSC, Return of spontaneous circulation; QUAL, qualitative; QUAN, quantitative

**Table 3:** Additional study characteristics

Study	Study design/method	Emergency healthcare system	Principles regarding termination of resuscitation	Ethical aspects and approvals
Anderson et al	Interviews	Intensive Care Paramedics are the definitive PRPs attending most community cardiac arrests, although basic life support responders – often the New Zealand Fire Service – are commonly first at the scene. Medical advisors can be consulted by phone, but doctors rarely attend emergency callouts	N/A	Ethical approval by University of Auckland Human Ethics Committee (Reference No 016147)
Anderson et al	Semi-structured interviews	Emergency response is provided by paid and volunteer ambulance personnel of varying practice levels (First Responder, EMT, Paramedic, Intensive Care Paramedic)	EMT level and above are authorized to commence, continue, withhold or terminate resuscitation and verify the death per national ambulance clinical guidelines	Ethical approval by University of Auckland Human Ethics Committee (Reference No 016147)
Brandling et al	Focus groups with case vignettes	N/A	There is well-established UK clinical practice guidance, based on the 2015 UK Resuscitation Council Guidelines that indicates when EMS providers (paramedics) should commence and cease resuscitation in OHCA. These guidelines are used by EMS providers (paramedics) to make decisions on whether to commence ALS and whether to carry on or cease ALS in OHCA	No ethical approval. Participants signed consent forms before participants
Bremer et al	face-to-face interviews	The ambulance teams include at least one registered nurse, often a specialist in emergency, intensive, or anaesthesia care	NA	No ethical approval. Conforms to ethical principles in medical research involving human subjects as outlined in the Declaration of Helsinki. Written consent was obtained from study participants
Davey et al	An exploratory, interpretive study using Vx, a web-based ethical decision-making tool	New Zealand paramedics operate under three scopes of practice: EMT, intermediate life support, and intensive care paramedic. There are two land-based ambulance services and 21 air ambulances	N/A	Ethical approval by AUTEC, AUT University’s Ethics Committee
Karlsson et al	Interviews	Ambulance personnel in Sweden may hold one of three professional competence certifications: basic EMT with a vocational education or equivalent registered nurse with a 3-year bachelor degree, or specialist ambulance nurse with a 3-year bachelor degree and an additional 1-year specialist education at a university with a focus on pre-hospital care	N/A	No ethical approval. Followed the ethical principles according to the Swedish Research Council. Written and oral approval was obtained from the management officers of the ambulance service organisation. Participants were provided both oral and written information concerning the purpose of the study
Larsson et al	Semi-structured interviews	N/A	Physicians are authorized to commence, continue, withhold or terminate resuscitation	Ethical approval by The University Ethics Committee at Luleå University of Technology. Written and oral consent was obtained from participants
Leemeyer et al	Semi-structured interviews, focus group	Prehospital EMS in The Netherlands is primarily provided by ground ambulance crews staffed with a driver and a certified nurse. There are no ambulance paramedics in the Netherlands. A HEMS team consists of a helicopter pilot, a board-certified physician (either trauma-surgeon or anesthesiologist), and a specialized ambulance or emergency room nurse	While HEMS physicians have the ultimate decisive authority from the moment HEMS are dispatched, many of the decisions around traumatic cardiac arrest (e.g., initiating resuscitation or not, thoracic decompression, etc.) will have to be made by ground EMS in the absence of a HEMS team	The study was exempted by the local Medical Research Ethics Committee. No information on consent from participants
Lord et al	Focus group interviews	N/A	N/A	Ethical approval by Monash University Human Research Ethics Committee and the Queensland University of Technology (QUT) Human Research Ethics Committee. No information on consent from participants
Naess et al	In-depth interviews	The EMS system in Oslo is a one-tiered centralized community-run system for a population of 470 000. Each response team consisted of two paramedics, one team also included an anesthesiologist	The personnel follow standing orders and do not need to contact a base station to obtain permission before initiating or discontinuing therapy	Ethical approval by the Regional Committee for Medical Research Ethics. Informed consent was verbal, as a signed consent was thought to put unnecessary pressure on the participants
Nordby et al	Naturalistic, semi-structured interviews and a cognitive-emotional, interpretive approach	N/A	Paramedics are authorized to make resuscitation decisions. Contact with medical specialists and other health personnel is limited, and the communication typically happens through a narrow interactive communication channel	All participants read and signed a form that explained the nature of the research. They also signed a statement that explained the aims and scope of the interviews, and that their participation was voluntary and based on informed consent
Nurok et al	Fieldwork (Observations and informal interviews)	In Paris, pre-hospital emergency work is performed by physician-led mobile intensive care unit teams containing a minimum of a consultant physician and ambulance driver. In addition, teams usually included a senior medical student, resident, and nurse anesthetist Given that emergency providers in the United States are supposed to provide minimal on-scene treatment, pre-hospital emergency providers are not as highly educated as they are in France. Calls result in teams of either Paramedics or First Aid providers being sent depending on the estimated severity of a case. These teams are often assisted by the Fire Department. Teams are supposed to follow strict protocols which stipulate action to be undertaken for any case. In New York, paramedics were able to call a physician for advice or authorization for certain treatments	N/A	No information on ethical approval or ethical aspects
*Quantitative studies*				
Druwé et al	Survey	Doctors, nurses, and EMTs/paramedics working in emergency departments and the prehospital setting	N/A	Conducted in accordance with the Declaration of Helsinki. Unless informed consent was not required, the study was approved by the Institutional Review Board of all participating countries
Druwé et al	Survey	N/A	N/A	Conducted in accordance with the Declaration of Helsinki. Unless informed consent was not required, the study was approved by the Institutional Review Board of all participating countries
Haidar et al	Survey	This is a setting with an underdeveloped EMS system that lacks national standards for prehospital care EMS volunteers in Lebanon get their training regardless of how far they reached in school and are not required to have any background in health education	N/A	No information on ethical approval or ethical aspects
Hick et al	Survey	The metropolitan area has a two-tiered dual response. Two-paramedic ALS ambulances respond to all ALS calls. In addition to the paramedic ALS unit, an AED (automated external defibrillator)-equipped first-responder unit is dispatched by the 911operator	Once radio contact is established, further therapy and disposition of the patient are determined by the paramedics in consultation with the physician; such decisions may include field termination of resuscitation, if appropriate	No information on ethical approval or ethical aspects
Johnson et al	Survey	N/A	In New Mexico, EMTs are taught to initiate CPR according to American Heart Association standards. EMTs are to initiate resuscitation on all patients unless decapitation, decomposition, or liver/rigor mortis exist	This study was reviewed by the University of New Mexico School of Medicine Human Research Review Committee
Leibold et al	Survey	N/A	Paramedics are prohibited from withholding resuscitation by German jurisdiction and therefore are obligated to initiate full resuscitation of patients with no vital signs, although they can of course express their opinions toward the physician's decision-making if a physician is present Only the presence of severe injuries, which are not compatible with life and/or definite signs of death (e.g., livor mortis), legally absolve paramedics from withholding resuscitation	No ethical approval. Followed the Declaration of Helsinki
Meyer et al	Survey	Emergency physicians work on doctor-equipped ambulances	N/A	No information on ethical approval or ethical aspects
Mohr et al	Survey	N/A	Physician-staffed emergency medicine enables the emergency physician to decide on the termination of resuscitative efforts and to pronounce someone dead in the field	No ethical approval. The questionnaire was confidential and anonymous. The participants were informed about the objective of the study
Navalpotro-Pascual et al	Survey	N/A	N/A	No information on ethical approval or ethical aspects
Sam et al	Survey	N/A	N/A	Approval was obtained from the North Shore—LIJ Health System institutional review board. Participants were anonymised
Sherbino et al	Survey	This system is under the control of a medical director, who provides offline quality assurance without online medical delegation Offline medical control is remote from the point of care (e.g., chart review or delegation by protocol). Online medical control refers to medical delegation over the phone at the time of patient contact	EMT-Ds are not required to initiate the resuscitation of a person with absent vital signs in the setting of decapitation, rigor mortis, or body decomposition	
Stone et al	Survey	EMS is provided by the Los Angeles Fire Department, which has 3586 firefighters, of whom 767 are paramedics and 2819 are EMT-D Denver has 128 EMT paramedics and 850 firefighter EMT-basics in a two-tiered system in which firefighter EMTs are the first responders and dual, hospital-based, paramedic ambulances are dispersed as the second tier. The firefighter EMT-basics are certified to use defibrillators	In almost all of the EMS systems in the United States, the initiation of resuscitation is mandatory in the absence of (1) a physician on scene superseding paramedic protocols; (2) clinical signs of irreversible death; or (3) a state-approved written DNR directive	No information on ethical approval or ethical aspects
Tataris et al	Survey	The Chicago EMS System is a regional collaborative of hospital-based EMS physicians and nurses that provide medical oversight for EMS provider agencies in the City of Chicago. The largest provider agency in the Chicago EMS System is the Chicago fire department, which provides exclusive emergency response for 9–1-1 calls in the City of Chicago Emergency calls for OHCA identified at the point of emergency medical dispatch result in the tiered response of a 4-person basic or ALS fire suppression company; a 2-person ALS transport ambulance, and a paramedic field chief	The Chicago EMS System has had an out-of-hospital TOR protocol since 1995, although very few victims of OHCA underwent termination in the out-of-hospital setting despite meeting TOR criteria	No information on ethical approval or ethical aspects
*Mixed-method studies*				
de Graaf et al	Registry data, semi-structured interviews	N/A	In the Netherlands, paramedics are legally allowed to make TOR decisions in the pre-hospital setting without consulting a physician. It is rarely documented which factors contribute to the decision to transport or terminate resuscitation of a patient when resuscitation appears to be unsuccessful	Ethical approval by The Medical Ethics Review Board of the Amsterdam UMC, Academic Medical Center. Written consent was obtained from participants
Waldrop et al	The survey, in-depth interviews	N/A	In the absence of a DNR order, prehospital providers have often been compelled to begin and continue resuscitation unless or until it is certain that the situation was futile and they have faced conflict when caregivers objected Most EMS companies have had protocols in place that allow their prehospital providers to conduct TOR	The study protocols were approved by the University at Buffalo Social and Behavioral Institutional Review Board. All participations were voluntary and anonymous

N/A = not available, EMT = Emergency medical technician, EMT-D = EMTs with defibrillation skills, OHCA = out-of-hospital cardiac arrest, EMS = Emergency medical service, ALS = advanced life support, QUAL = qualitative, QUAN = quantitative, DNR = do-not-resuscitate, CPR = cardiopulmonary resuscitation, HEMS = Helicopter Emergency Medical Service, PRP = Prehospital resuscitation provider

### Quality assessment

For quality appraisal scores, see Table 2. The MMAT quality score varied among the studies. The qualitative papers had a median score of 100% (range 0–100%), the quantitative papers had a median score of 80% (range 60–100%), and the mixed methods papers had a median score of 60% (range 20–100%).

### Descriptions of non-medical factors influencing prehospital providers’ decision-making

**Fig. 2:**
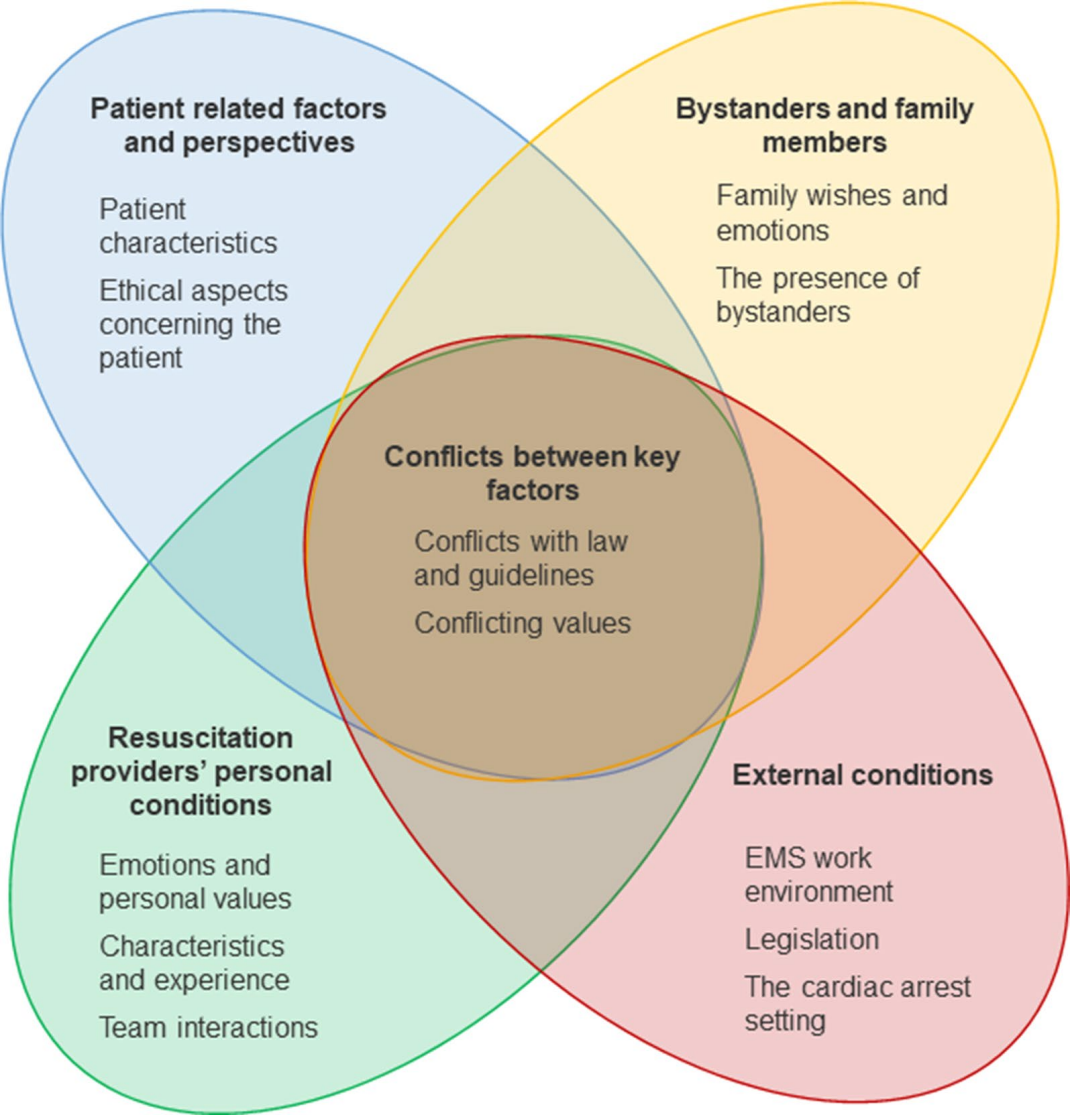
A visual presentation of the identified themes and their relations

Our analysis revealed five main themes and 13 subthemes. See Fig. 2. Both qualitative and quantitative studies covered all themes, but we identified differences between qualitative and quantitative findings regarding emotions, values, and personal beliefs of providers and their influence on decision-making. These were found mainly in qualitative studies. Quantitative studies mostly concerned the patients’ age, the providers’ characteristics and experience, and advance directives. All five main themes were covered by studies from various geographical origin. See Table 4 for an overview of factors and how they influence decision-making.

**Table 4:** Non-medical factors and their influence on decision-making

Themes and subthemes			Non-medical factors influencing initiation/continuation of resuscitation^a^	Non-medical factors influencing withhold/termination of resuscitation^a^	Non-medical factors influencing decision-making non-specifically^a^	Non-medical factors explicitly mentioned as NOT influencing decision-making^a^
*Applying patient-related factors*						
Patient characteristics	Age	QUAL	Young age [27, 29, 31, 32]		Age in general [5, 23]	
		QUAN	28.8% (n = 288) would almost always perform CPR on the young patient [42]	Perception of inappropriate CPR was significantly higher for cardiac arrests in patients older than 79 years of age (OR = 2.9 [95% CI 2.18–3.96]; P < .0001) [34]	Age in general [41]	
	Social status	QUAL	Low social value if treated by a novice PRP (for practice purposes) [31]		Being aware of social status, but not being influenced [29]	
Ethical aspects	Perceived prognosis	QUAL		Expected low QoL *after* resuscitation [27, 30] Subjective assessed worn-out or morbid appearance [22] Perception of risk of post-resuscitation major impairment [5, 30]		
		QUAN		21.3% (n = 45) expressed concern for the patients (incl. quality of life) in a system, where EMTs were not allowed to terminate resuscitation [44] 2% (n = 8) mentioned expected low QoL *after* resuscitation in additional free-text answers [41] Perception of inappropriate CPR was significantly higher for cardiac arrests in patients whose first physical impression was rated “bad” to “poor” by the reporting clinician (OR = 3.7 [95% CI 2.78–4.94]; P < .0001 and OR = 3.5 [95% CI 2.36–5.05]; P < .0001, respectively) [34]		
	Dignity	QUAL		Allowing the patient to die “a natural death” or “die with dignity” [25, 29, 44]		
		QUAN		21.3 (n = 45) expressed concern for the patients (incl. allowing the patient to “die with dignity”) in a system, where EMTs were not allowed to terminate resuscitation [44]		
	Patient’s wishes	QUAL	Lack of DNACPR [28]	Presence of DNACPR [23]		The patient’s wishes were absent from many participants decision-making processes [22]
		QUAN		95% (n = 223) of paramedics believed “strongly” or “somewhat” that prehospital providers should honour written ADs in the field [45] About 74.5% (n = 320) would not resuscitate a dying patient who has an advance directive or DNACPR order, were they given legal certainty. [39] 73.7% (n = 176) felt confident when there was a DNACPR order, and they did not initiate resuscitation [33] 48.8% (n = 481) would not start CPR in the presence of DNACPR orders as presented by the family [42] 43% (n = 99) of PRPs would perform CPR instead of wasting time to locate a DNACPR [43] 1% (n = 4) mentioned the availability of a living-will declaration or the presumed will to live in additional free-text answers [41]		
	Patient’s best interests	QUAL	Giving the patient “the benefit of the doubt” [23]	Perceiving termination to be “the patient’s best interests” [30]	Evaluating the patient’s best interests [22]	
*Involving of and involvement by bystanders and family members*						
Family wishes and emotions	Family wishes	QUAL	Begging and pleading for continuation [24, 29] Family religion dictating continuation [32]	Family members fearing permanent vegetative state [29]	Family wishes [22] Involving family as the patient proxy [29]	
		QUAN	8% (n = 3) continued because family did not accept termination [37]	59% (n = 138) would honour family wishes [45] 10.8% (n = 108) would honour family wishes [42]	Involving family as the patient proxy [41]	
	Buying time for the family	QUAL	Giving the family time to realize the patient’s death/saying goodbye [5, 24] Showing the family that everything has been done [24] Perceived wishes of continuation [24, 29]	Eliminating false hope [33]		
		QUAN			Unnecessary emotional trauma [44]	
	Coping with the family’s emotions	QUAL	Avoiding dealing with family’s emotions [24, 29] Perception of better family support in-hospitally [32]	Continuation of CPR creates hope [24]		
		QUAN	0,4% (n = 1) stated cultural barriers lead to transportation [37]	45.29% (n = 108) were comfortable with the termination of resuscitation when they knew that death was imminent [33]	52% (n = 1200) were uncomfortable with terminating resuscitation [46] 69.1% (n = 165) were comfortable dealing with a family’s emotional response to death [33]	
	Identifying with the family	QUAL	Continuation of futile CPR [24]			
The presence of bystanders	Meeting expectations	QUAL	CPR for “show” [24, 27] Presence of bystanders/relatives [29, 32]		Expectations and perceptions of bystanders [8]	
		QUAN	70.1% (n = 180) stated bystanders’ reactions as a reason for prolonging CPR [36]			
	Respecting bystander efforts	QUAL	Acknowledging bystander CPR [5, 29]			
		QUAN	31.7% (n = 317) would initiate/continue obvious futile CPR to acknowledge bystander CPR [42] 26.6% (n = 266) would continue for teaching purposes [42]			
*Personal conditions have an impact*						
Characteristics and experience of PRPs	PRPs’ age	QUAN	Younger PRPs were more inclined to initiate CPR [42]	Older clinician’s age was negatively associated with perceptions of appropriate CPR [34]		No association between age and paramedics’ attitudes toward withholding resuscitation attempts based on written or verbal ADs [45]
	PRPs’ gender	QUAL			PRPs gender influence decision-making [31]	
		QUAN	Women were more inclined to initiate or continue CPR in patients with terminal illness [42] Men are more inclined to initiate or continue CPR for teaching purposes [42]	Male providers were significantly more likely to report believing that resuscitation ought to be terminated in case of advanced directives than female providers (42.8% vs. 25.3%) [36]		
	PRPs' level of education	QUAL			Level of education influences decision-making [23]	
		QUAN	Out-of-hospital nurses showed a greater tendency to perform CPR in situations of terminal illness or poor basal condition, and also to perform CPR even when not indicated [42]	Education on the appropriateness of CPR [34]	Paramedics were more likely to be troubled by terminating resuscitation than EMTs (P = .019) [38]	
	Type of daily work	QUAL			A notable difference between their responses relating to the team they worked in and the type of work they encountered daily [23]	
		QUAN	Surgeons [40]	Surgeons [40]		No significant associations were found between the profession of the clinician and the perception of inappropriate CPR [34]
	Experience	QUAL	Inexperience [22, 32] Experiences with successful resuscitation [29, 32]	Level of experience, with experienced PRPs more inclined to terminate resuscitation [8, 22]	Experience may influence decision-making [23, 30, 31] Experience from previous cases [5, 29]	
		QUAN	Inexperienced [42]	Association between experience and believing “death is a part of life" (P = 0.032), “withholding resuscitation is resuscitation ethical" (P = 0.048) and that one should "not resuscitate a patient who holds a DNAR order" (P = 0.002) [39] EMS professionals who had more than 16 years of experience were more comfortable honouring the MOLST (83%) than those with 6 years or less (55%, P < .007) [43]	Experience [38]	There was no association between years of EMT-D service and willingness to honour a DNACPR order (p = 0.47) [44] No association between years of experience, or personal EOL decision-making experience and paramedics’ attitudes toward withholding resuscitation attempts based on DNACPRs [45] Results showed tendencies of PRPs with a higher level of experience may shorten the duration of unsuccessful resuscitative efforts, but this was not significant [41]
Emotions and personal values	Uncertainty	QUAL	Making sure nothing is missed [22] Uncertainty [8, 22] Starting CPR immediately to save time [29] Requiring verifiable information [8]		Uncertainty [24, 31] Clinical uncertainty [28]	
		QUAN	33% (n = 762) feared terminating the resuscitation too early [46]			
	Personal values	QUAL	PRP’s religion [32] Heroic value [31] Responsibility [29]	Termination of resuscitation of a patient who might face a quality of life they would consider unacceptable for themselves [8]	Interpersonal factors influence the application of formal guidelines [24]	
		QUAN				There was no significant association between religiosity and the following questions: "Is death a part of life?" (P = 0.07), "Is every human life worth living, no matter the circumstances?" (P = 0.06), "Would you resuscitate a patient who holds an advanced directive that clearly states he/she does not want to be resuscitated?" (P = 0.64) [39] No association between perception of appropriate CPR and religiosity (p = 0.61) [34]
	Fear of consequences	QUAL	Fear of legal issues or criticism [23, 29, 32] Official complaints [27]		Fear of working outside practice guidelines [28]	
		QUAN	Fear of legal issues or criticism [39]		Fear of legal issues or criticism [44]	
Team interaction	Team interaction	QUAL	When team members had conflicting opinions, the opinion to transport generally prevailed over the opinion to terminate on-scene [5, 32]	Consulting with a superior [27] Getting advice from others [22]	Team agreement may influence decision-making [26] Consulting with a superior [23, 30, 32] Crew composition [23]	
*Being influenced by external factors*						
EMS work environment	Emergency Medical System	QUAL	The reputation of the EMS system [31] System-related pressure to save lives no matter what [30]		Organizational support [23] The reputation of the EMS system [29]	
		QUAN			Concerns about inappropriate resource utilization if all patients are sought resuscitated [44]	
	Training purposes	QUAL	Training purposes [29, 31]			
		QUAN	26.6% (n = 266) indicated to initiate or continue resuscitation for training purposes “sometimes”, “often” or “almost always” [42]			
	Provider fatigue	QUAL	Provider fatigue at the end of a shift [23]			
	Crew safety	QUAL	Crew safety [8, 29, 32]			
		QUAN	86% (n = 1985) indicated scene safety as a barrier to terminate resuscitation [46]		Feeling threatened by family in case of termination [46]	Scene safety was not cited as an issue [38]
	Area of service	QUAN		Rural areas [36]		
Legislation	Formal guidance	QUAL	Uncertainty about legislation [23]		Some PRPs were guided by the law [25] Some PRPs felt conflict about withholding resuscitation and lacked confidence in decision making about TOR [33]	
		QUAN	6.6% (n = 36) CPR attempts were undertaken despite the presence of a known written do not attempt resuscitation (DNAR) decision. Of these, 38.9% (n = 14) clinicians considered the CPR appropriate, 25.0% (n = 9) were uncertain about its appropriateness, and 36.1% (n = 13) considered this inappropriate [34]	28.9% (n = 69) felt conflicted about what to do when there was a DNACPR, and the family called 911, and 41.4% (n = 99) felt conflicted when there was no DNACPR and the family asked them not to resuscitate [33]	Some PRPs were guided by the law [39] Only 9.8% (n = 42) think that they are competent to handle advanced directives [39]	
The arrest setting	Location of arrest	QUAL		Settings that were associated with high mortality and morbidity [22]		
		QUAN			Location of arrest [37]	
	The environment	QUAL	Weather conditions [32]		Environmental conditions [8]	
		QUAN	Weather conditions [37]			
	Logistics	QUAL			Logistical limitations [8] Long distances [32]	
*Navigating conflicts in the area of tension between key factors*						
Conflicts with the law and guidelines	Legal and guidelines	QUAL	Moral decisions were overridden by protocol [28]	Deviation from the guidelines to respect the patient’s dignity [26]	Balancing patient’s wishes and legislation [23]	
		QUAN	76.6% (n = 328) of the paramedics stated that they had no legal latitude in withholding resuscitation in a dying and terminally ill patient [39] 63% (n = 148) would disregard the DNACPR order and initiate resuscitation [44]			
Conflicting values	Family wishes	QUAL	In case of disagreement between family members regarding the DNACPR status of the patient, the resuscitation was continued [32]		Family wishes vs. patient’s rights [23, 25, 26] Family wishes vs. resuscitations providers personal values [29]	
		QUAN			24.4% (n = 58) experienced conflicts between patient and family [33]	
	The duty to save lives	QUAL	The conflict between own moral beliefs and system expectations [30]		Balancing duty and values [24]	
	Team interaction	QUAL	Conflicting values in the resuscitation team [8] Conflicting interpersonal factors [23]	HEMS personnel believed ambulance nurses not initiating resuscitation in patients where they felt this would have been appropriate [5]	Conflicting values in the resuscitation team [31]	
	Lack of information	QUAL	PRPs would start resuscitation regardless of this in almost all situations and rather collect additional information during resuscitation to support further decision making [5]		Incomplete or conflicting information [8]	

QUAL, Qualitative; QUAN, Quantitative; QoL, quality of life; DNACPR, do not attempt CPR; HEMS, Helicopter Emergency Medical Service; EMS, Emergency Medical System; PRP: Professional Resuscitation Provider

^a^In studies where the percentage of participants in a given group was provided, the number of participants (n) was calculated by hand. Some quantitative studies did not provide specific percentages nor the number of patients, and in these cases, only the corresponding narrative theme has been provided

#### Applying patient-related factors and perspectives

##### Patient characteristics

Patient age formed a part of the decision-making [41]. In younger patients, some providers would almost always perform resuscitation [42] and continue resuscitation for longer [8, 27, 29, 31, 32]. Old age in itself was not described as a reason to cease treatment, but young age was reported to be a reason to initiate resuscitation [29] and treat the patient more aggressively [31]. The providers felt a greater weight of responsibility and emotional burden when terminating resuscitation in younger patients [8]. In line with this finding, patients’ age above 79 years increased the perception of inappropriate resuscitation attempts [35].

Social status was considered in decision-making [29, 31]. A patient’s social status was thus explicitly reported not to influence resuscitation [29] while another study noted the patient’s social value did influence the decision-making [31]. Social value was described as certain personal patient attributes that are highly valued by an ensemble of members of a society [31]. Patient gender was not mentioned in any studies.

*Ethical reflections concerning the patient’s wishes and quality of life* Perceptions of the patient’s prognosis and expected quality of life were described as influencing decisions [5, 22, 27, 30, 34, 41, 44]. The subjective assessment of morbidity or the patient’s clinical presentation tended to dominate decision-making over chronological age, explained by the fact that exact age and known comorbidities are often not known prehospitally [22]. The perception that resuscitation was inappropriate was significantly higher in patients where the providers found the first physical impression of the patient to be “poor” or “bad”. Providers mentioned the quality of life [27, 30], where an expected low quality of life in case of successful resuscitation would make providers question the treatment. When providers were legally obliged to initiate resuscitation, they described having concerns about the patient’s expected quality of life after resuscitation [44].

In some studies, patient wishes and advance directives were important factors in decision-making [23, 28, 33], while others reported a lack of consideration for patient wishes and formal documentation [22]. In some studies, the majority of participants would honour an advance directive [33, 39, 45]. In other studies, half of the participants would not honour an advance directive [42, 43]. Honouring an advance directive could be influenced by the age of the patient and the gender and experience of the provider [36, 39, 43]. Providers would most likely seek documentation of an advance directive if they were male or more experienced or if the patient was 60 years or older [39, 43].

Some studies highlighted concern for the patient’s best interests [22, 30, 47], either by discontinuing resuscitation in patients with a low expected quality of life in case of survival [30] or by giving the patient the benefit of the doubt continuing resuscitation for a longer period [23]. The patient’s best interests were not evaluated exclusively through advance directives expressing the patient’s wishes, but rather by conferring with the family and crewmembers [22].

Some providers believed that a seriously ill and old patient had the right to die a “natural death” or die with dignity [29]. Dignity was not only described as upholding the patient’s wishes but also the act of avoiding apparently futile procedures [25]. Providers highlighted the right to a dignified death [25] or to die without interference [29] and were concerned with the patients’ dignity in cases where they could not legally terminate resuscitation [44].

#### Involving and involvement of bystanders and family members

##### Family wishes and emotions

The patient’s family and their wishes, emotions, and/or expectations could pressure providers to the initiation, continuation [24, 29, 30, 32], and termination [29] of resuscitation. Some providers mentioned that the family’s religion could lead to pressure to continue obviously futile resuscitation when according to the religious beliefs, everything possible had to be done including transportation to the hospital [32]. In cases where the family wished for termination, the providers described the family as expressing fear of suffering or a permanent vegetative state for the patient [29]. Some providers would not follow family wishes [42]. Others believed family members’ verbal wishes should be honoured [45]. Family wishes were mentioned as a reason for not complying with the guidelines [37, 46]. The family’s despair led them to beg or plead for the continuation of resuscitation—a request that the providers often complied with [24, 29], but the emotions of the families also increased the providers’ uncertainty if their decision went against the expressed wishes of the family [24, 30]. Some providers voluntarily involved the relatives in the decision-making process to obtain information or to aid the families in their mourning [29, 41].

Some providers continued resuscitation to accommodate the perceived needs of the family members [5, 24, 29]. This included continuing resuscitation to ensure that everything possible had been done to save the patient [24], but also to give the family time to realise the patient’s imminent death [5]. The family’s acceptance of a severely ill or older patient’s death could make providers more likely to withhold resuscitation [29]. On the other hand, providers feared increasing unrealistic expectations of survival if they continued resuscitation to allow the family to say goodbye [24, 33, 44]. Continuation of resuscitation and transportation to the hospital was the providers' way to deal with their inadequacy in meeting the family’s needs [24] and avoid facing the relatives with information that resuscitation was futile [29]. The in-hospital environment was perceived by providers as making it easier for the family to accept termination of resuscitation [32]. Close identification with the family made some providers more subjective in their decision-making and lead to the continuation of futile resuscitation [24]. Cultural barriers and an inability to identify with the emotional response of the family could lead to transportation of the patient to avoid confrontation [37].

##### The presence of bystanders

The presence of other bystanders than the family could influence the providers to continue resuscitation [36] to show that something had been done to attempt to save the patient’s life [24] or for providers to look their best in front of spectators [27]. The providers in one study displayed frustration with the bystanders’ unrealistic expectations [8]. These high expectations could influence the providers to continue resuscitation and transport the patient [29, 32]. When bystanders had initiated resuscitation, providers felt an obligation to continue resuscitation to respect the resuscitation attempt [5], to assure bystanders that their effort was important for a good outcome [29], and encourage them to do resuscitation again in a similar situation [42].

#### The personal conditions of providers

##### Provider’s characteristics and experience

Pre-existing conditions among others, length of service, type of daily work, or training were described as influencing decision-making [23]. Surgeons could be more likely to initiate resuscitation compared to anaesthesiologists and general practitioners [40]. One other study, however, found no association between profession and the perception of the appropriateness of resuscitation [34]. The level of training was associated with doubts concerning withholding resuscitation with paramedics being more likely to be troubled than EMTs [38]. Providers who had received specific training were more inclined to find resuscitation appropriate [34] and providers with palliative training found it more reasonable to resuscitate dying and terminal patients [39].

The provider’s gender and age was described as influencing decisions [31, 36, 42]. One study did not find an association between age and withholding resuscitation [45]. Personal experience and years of service made the providers more confident in their decision-making [8, 22, 23, 31, 32, 38, 39, 41–43]. Two papers did not find an association between experience and honouring a do not attempt resuscitation order (DNACPR) [44, 45]. Some províders described that specific experiences had influenced their decision-making [5, 29, 30], e.g. receiving flowers or letters from elderly patients who survived a cardiac arrest, and made them more prone to initiate resuscitation despite old age [29].

##### Emotions and personal values

The providers described an uncertainty in decision-making [8, 22, 24, 28, 29, 31, 46] in unexpected situations [8]. This was often described in situations with a lack of information [22, 31]. Uncertainty could lead to prolonged resuscitation [8, 46]. providers feared overlooking information that was important for the treatment and not having done everything possible to save the patient [22, 29]. Others feared legal consequences, criticism, or disciplinary procedures if they did not initiate or continue resuscitation [23, 27–29, 32, 44].

The providers acknowledged that their emotions, beliefs, and values provided a lens through which they viewed the patient’s and family’s emotions and reactions and that this might influence the decision-making [8]. Some providers noted that their own religious beliefs influenced their decision-making [33] while two studies did not support this association between religion and decision-making [34, 39].

##### Team interaction

Agreement within the team and the respect of other team members’ opinions were important [5, 23, 26, 32]. Team members who wanted to continue resuscitation prevailed over those who would not [5, 22, 23, 26, 27, 30, 32]. Several studies reported that non-physician providers contacted a physician to ensure everything had been done before terminating resuscitation [22, 23, 27, 30, 32]. Some providers mentioned that the composition of the prehospital crew (e.g., type of work, experience, etc.) influenced the decision-making [23].

#### Being influenced by external factors

##### Emergency medical service system and work environment

The providers described being influenced by the level of support from their employer [23] and the emergency healthcare system’s reputation [29–31]. The providers wished to contribute to a positive EMS reputation and sometimes adjusted their actions according to the perceived wishes of bystanders [29].

Some providers continued resuscitation for teaching or training purposes [29, 31, 42]. One study found provider fatigue as an influence referring to the long and odd working hours in the EMS [23], which led to deciding faster to transport or continuing longer at the scene to avoid an accusation of not having done enough [23]. Threats from the family could lead providers to commence or continue resuscitation and transport the patient [8, 29, 32, 46]. Scene safety was not cited as an issue in one study [38]. Rural versus urban area of service was a factor in one study with providers in rural areas more likely to terminate resuscitation compared to providers in urban areas [36].

##### Legislation and official guidelines

Providers found that legislation and official guidelines influenced decisions [35, 39]. Some found them helpful [25], while others felt uncertain about legal obligations [23, 33]. In one study, only 9.8% of providers felt competent to handle advance directives [39], while in another study, 73.7% felt confident terminating resuscitation when a DNACPR was present [33].

##### The cardiac arrest setting

The providers reported that the prehospital setting influenced decision-making [8, 22, 32, 37]. Specific areas with high mortality and morbidity, such as nursing homes and low socioeconomic areas, influenced treatment [22] as did the location of arrest e.g. public place [37]. Weather conditions and other environmental conditions e.g., cramped, dark places could influence resuscitation [8, 32, 37]. Logistical limitations e.g. difficulties of doing CPR during transport were mentioned in one study [8], while another study did not find the distance to the hospital to influence the decision-making [32].

#### Navigating conflicts

*Ethical conflicts between guidelines, legislation, and beliefs* The providers experienced conflicts between the law, the guidelines, and the patient’s wishes [23, 26] [44]. Specifically, they mentioned the lack of formal documentation in situations where family members stated that the patient did not wish to be resuscitated [23] and believed that ethically correct decisions sometimes resulted in deviations from guidelines or legislation [26, 28].

##### Conflicting values

The providers described various values and beliefs that created conflicts during decision-making [33]. The family could express different opinions on resuscitation than those noted by the patient in advance directives [23, 25, 26, 29, 32]. To avoid conflicts, providers would continue or transport the patient during resuscitation [32]. Providers experienced conflicts between their own values and the expectations from the legislative system [24, 30]. They feared the negative consequences if they did not transport the patient [30] and felt a conflict between the perceived legal obligation to save lives contrasting with the best interest of the patient [24].

The providers' values could conflict within the resuscitation team [5, 8, 23, 31]. In all studies, disagreements arose over the appropriate level of aggressiveness of treatment. The providers balanced futile care against a well-founded decision and described conflicts and challenges in choosing whether to initiate or continue resuscitation in cases where information was lacking [5, 8]. For example, almost all participants in one study would initiate resuscitation despite the feeling of providing futile care [5].

## Discussion

In this systematic review concerning non-medical considerations in decision-making during prehospital resuscitation, we identified various themes influencing prehospital providers' decision-making, including patient-, family-, and provider-related factors as well as external factors such as legislation. Furthermore, we identified conflicts occurring between the influences coming from various actors, and differences between findings in qualitative and quantitative studies. Our findings underline the importance of contributions from both study methodologies to gain a better understanding of the decision-making process and the various influences. The study aims and the topics in the included studies varied from end-of-life to sudden cardiac death, which may explain variation in study results. However, this is a reflection of the wide spectrum of cardiac arrest scenarios that providers attend.

Non-medical factors are diverse. We identified several areas of potential improvement in out-of-hospital cardiac arrest decision-making: First, several factors, which should ideally be *avoided*, were described as part of decision-making. Examples of this include social status, the location of the cardiac arrest, and provider bias which risk challenging the bioethical principle of justice and hence should be avoided in decision-making [48]. However, complete avoidance of such factors may be difficult to attain. Instead, efforts to encourage providers to consciously reflect on non-medical factors in decision-making may be helpful.

Secondly, some non-medical factors, which should be *included*, were not always considered. One of the most researched non-medical factors were DNACPRs. Interestingly, studies on DNACPRs and their use in decision-making showed diverging results. The handling of advance directives differed and we found diversity in individual studies where participants were divided almost equally between their beliefs on DNACPR and their handling in practice [41–43]. Current guidelines underline that information about the patient’s wishes and values on resuscitation should be sought and included in resuscitation decision-making [3], but our findings suggest that providers find it difficult to do this in practice. Up to 10% of out-of-hospital cardiac arrests involve DNACPRs and in these cases, conflicts can occur between the patient’s family and providers [49]. Most of the included studies were conducted in a prehospital setting without a universal termination of resuscitation rule [50], but in the few studies where these rules were implemented, non-medical factors were described as influencing treatment and could also collide with the provider’s personal beliefs [32]. These conflicts of priorities, together with ethical conflicts, conflicts of expectations, fear of litigation and uncertainties, can influence decision making in unfortunate ways and furthermore cause moral distress in healthcare professionals [51]. Interventions to support providers’ prehospital resuscitation decision-making could target these situations, and future studies could shed further light on the conflicting factors in resuscitation and the dilemmas in providers’ coping.

### Limitations

Our study has several limitations. The study aims and the topics in the included studies varied from end-of-life to sudden cardiac death. Thus, variation in study results may have occurred. The included studies are from a wide range of countries and have different study aims, EMS structure, and publication years. This precluded a meta-analysis. Furthermore, non-medical factors are difficult to define. We may thus have overlooked some studies. The resuscitation guidelines are updated every 5 years. This may induce changes in the resuscitation practices and thus contribute to limitations in our study. However, we found representations of all themes in both newer and older studies. As clinical decision-making including ethical considerations is a sensitive area, response bias may have occurred. Three qualitative studies rated as high quality with the critical appraisal tool [5, 30, 31], did not state their methodological orientation nor supported the analysis process with any references. As qualitative studies explore the content and meaning of empirical data, not precisely stating the methodological orientation and referencing the theoretical approach makes it difficult to decipher how the authors analysed their data. Thus, it should be noted that high reporting rates according to MMAT do not necessarily equate to high‐quality studies.

## Conclusion

When deciding whether to initiate, continue, terminate, or refrain from resuscitation, prehospital resuscitation providers are influenced by a plethora of factors of which some are not strictly medically related. The providers report that patient- and family-related factors influence their decision-making process. They further report that they are influenced by personal conditions and external factors. Additionally, the providers may experience that conflicts between various factors complicate decision-making. Future research should consider non-medical factors and their role in decision-making.

## Supplementary Information

**Additional file 1**. PRISMA checklist.

**Additional file 2**. Full search strategy.

**Additional file 3**. List of search terms.

**Additional file 4**. Overview of the synthesis process.

## Data Availability

Data sharing is not applicable to this article as no datasets were generated or analysed during the current study.
