# Supplementary material for: Non-medical factors in prehospital resuscitation decision-making: a mixed-methods systematic review
Source: Scand J Trauma Resusc Emerg Med. 2022 Mar 28;30:24. doi: 10.1186/s13049-022-01004-6 (PMC8962561; doi:10.1186/s13049-022-01004-6)
Supplement: Supplementary file 2 — Additional file 2. Full search strategy. [file 13049_2022_1004_MOESM2_ESM.docx]

Non-medical factors in prehospital resuscitation decision-making: A systematic review

**Search strategy**

Final search: June 11^th^ 2021

| **Pubmed** | |
| --- | --- |
| #1 | ("Thinking"[Mesh] OR "Decision Making"[Mesh] OR "Clinical Decision-Making"[Mesh] OR "Judgment"[Mesh] OR "Problem Solving"[Mesh] OR "Withholding Treatment"[Mesh] OR "Resuscitation Orders"[Mesh] OR decision making OR decisions OR termination OR withhold OR judgment OR critical thinking OR decision process OR choice behaviour OR problem solving OR withholding treatment OR Withdraw OR Terminate OR Resuscitation orders OR medical futility OR Coping behaviour OR DNR OR Do-not-resuscitate OR advance directive OR living will) |
| #2 | ("Out-of-Hospital Cardiac Arrest"[Mesh] OR Out-of-hospital cardiac arrest OR out of hospital cardiac arrest OR out of hospital cardiac arrests OR cardiac arrest OR heart arrest OR cardiopulmonary resuscitation OR resuscitation OR CPR OHCA OR out-of-hospital cardiorespiratory arrest) |
| #3 | ("Emergency Responders"[Mesh] OR "Emergency Treatment"[Mesh] OR "Emergency Medicine"[Mesh] OR "Emergency Medical Technicians"[Mesh] OR "Emergency Medical Services"[Mesh] OR "Ambulances"[Mesh] OR Prehospital OR pre-hospital OR out-of-hospital OR out of hospital OR emergency health service OR emergency medical service OR EMS OR paramedic OR emergency medical technician OR EMT OR prehospital care OR emergency medical care OR emergency health care OR acute medical care OR acute care OR emergency medicine OR first responders OR rescue workers OR emergency personnel OR emergency preparedness OR emergency responders OR emergency treatment) |
| #4 | #1 AND #2 AND #3 |

| **CINAHL** | |
| --- | --- |
| #1 | ((MH "Thinking+") OR (MH "Critical Thinking") OR (MH "Decision Making, Clinical") OR (MH “Decision Making+”) OR (MH “Problem Solving”) OR (decisions OR decision-making OR withhold OR judgement OR critical thinking OR decision process OR choice behavior OR ethical decision making OR problem solving OR terminate OR resuscitation orders OR DNR OR do-not-resuscitate OR decide OR withdraw OR medical futility OR coping behavior OR advance directive OR living will)) |
| #2 | ((MH “Heart Arrest+”) OR (MH "Resuscitation") OR (MH "Resuscitation Orders") OR (MH "Resuscitation, Cardiopulmonary") OR (out of hospital cardiac arrest or ohca or cardiac arrest or heart arrest OR cardiopulmonary resuscitation OR resuscitation OR out-of-hospital heart arrest OR cardiorespiratory arrest)) |
| #3 | ((MH "Emergency Service+") OR (MH "Emergency Medical Technician Attitudes") OR (MH "Physicians, Emergency") OR (MH "Emergency Patients") OR (MH "Emergency Medicine") OR (MH "Emergency Medical Technicians") OR (MH "Emergency Treatment") OR (MH "Emergency Care+") OR (Prehospital OR pre-hospital OR out-of-hospital OR out of hospital OR emergency health service OR emergency medical service OR EMS OR paramedic OR emergency medical technician OR EMT OR prehospital care OR emergency medical care OR emergency health care OR acute medical care OR acute care OR advanced trauma life support care OR ATLS OR ATLSC OR emergency medicine OR first responders OR rescue workers OR emergency personnel OR emergency preparedness)) |
| #4 | #1 AND #2 AND #3 |

| **OVID databases (EMBASE, MEDLINE, PsycINFO combined)** | |
| --- | --- |
| #1 | exp decision making/ or thinking/ or problem solving/ or treatment withdrawal/ or (decisions or decision-making or withhold or judgement or critical thinking or decision process or choice behavior or ethical decision making or problem solving or terminate or resuscitation orders or DNR or do-not-resuscitate or decide or withdraw or medical futility or coping behavior or advance directive or living will).mp. [mp=title, abstract, heading word, drug trade name, original title, device manufacturer, drug manufacturer, device trade name, keyword, floating subheading word, candidate term word] |
| #2 | heart arrest/ or "out of hospital cardiac arrest"/ or resuscitation/ or (out of hospital cardiac arrest or ohca or cardiac arrest or heart arrest or cardiopulmonary resuscitation or resuscitation or out-of-hospital heart arrest or cardiorespiratory arrest).mp. [mp=title, abstract, heading word, drug trade name, original title, device manufacturer, drug manufacturer, device trade name, keyword, floating subheading word, candidate term word] |
| #3 | emergency care/ or emergency treatment/ or emergency health service/ or emergency medicine/ or emergency physician/ or emergency patient/ or (Prehospital or pre-hospital or out-of-hospital or out of hospital or emergency health service or emergency medical service or EMS or paramedic or emergency medical technician or EMT or prehospital care or emergency medical care or emergency health care or acute medical care or acute care or advanced trauma life support care or ATLS or ATLSC or emergency medicine or first responders or rescue workers or emergency personnel or emergency preparedness).mp. [mp=title, abstract, heading word, drug trade name, original title, device manufacturer, drug manufacturer, device trade name, keyword, floating subheading word, candidate term word] |
| #4 | #1 AND #2 AND #3 |
| #5 | 4 use emczd* |
| #6 | use medall* |
| #7 | exp Decision Making/ or Thinking/ or Judgment/ or Problem Solving/ or Medical Futility/ or Resuscitation Orders/ or  (decisions or decision-making or withhold or judgement or critical thinking or decision process or choice behavior or ethical decision making or problem solving or terminate or resuscitation orders or DNR or do-not-resuscitate or decide or withdraw or medical futility or coping behavior or advance directive or living will).mp. [mp=title, abstract, original title, name of substance word, subject heading word, floating sub-heading word, keyword heading word, organism supplementary concept word, protocol supplementary concept word, rare disease supplementary concept word, unique identifier, synonyms] |
| #8 | exp Heart Arrest/ or Out-of-Hospital Cardiac Arrest/ or Cardiopulmonary Resuscitation/ or Resuscitation/ OR (out of hospital cardiac arrest or ohca or cardiac arrest or heart arrest or cardiopulmonary resuscitation or resuscitation or out-of-hospital heart arrest or cardiorespiratory arrest).mp. [mp=title, abstract, original title, name of substance word, subject heading word, floating sub-heading word, keyword heading word, organism supplementary concept word, protocol supplementary concept word, rare disease supplementary concept word, unique identifier, synonyms] |
| #9 | exp Emergency Medical Services/ or Emergency Treatment/ or Emergency Medical Technicians/ or Emergency Medicine/ or (Prehospital or pre-hospital or out-of-hospital or out of hospital or emergency health service or emergency medical service or EMS or paramedic or emergency medical technician or EMT or prehospital care or emergency medical care or emergency health care or acute medical care or acute care or advanced trauma life support care or ATLS or ATLSC or emergency medicine or first responders or rescue workers or emergency personnel or emergency preparedness).mp. [mp=title, abstract, original title, name of substance word, subject heading word, floating sub-heading word, keyword heading word, organism supplementary concept word, protocol supplementary concept word, rare disease supplementary concept word, unique identifier, synonyms] |
| #10 | #7 AND #8 AND #9 |
| #11 | 10 use medall* |
| #12 | use psyh* |
| #13 | exp Decision Making/ or Thinking/ or Judgment/ or Problem Solving/ or Coping behaviour/ or "Clinical Judgment (Not Diagnosis)"/ or (decisions or decision-making or withhold or judgement or critical thinking or decision process or choice behavior or ethical decision making or problem solving or terminate or resuscitation orders or DNR or do-not-resuscitate or decide or withdraw or medical futility or coping behavior or advance directive or living will).mp. [mp=title, abstract, heading word, table of contents, key concepts, original title, tests & measures, mesh] |
| #14 | exp Heart disorders/ or CPR/ or (out of hospital cardiac arrest or ohca or cardiac arrest or heart arrest or cardiopulmonary resuscitation or resuscitation or out-of-hospital heart arrest or cardiorespiratory arrest).mp. [mp=title, abstract, heading word, table of contents, key concepts, original title, tests & measures, mesh] |
| #15 | exp Emergency Services/ or Emergency Medicine/ or Emergency Personnel/ or Emergency preparedness/ or Emergency management/ or (Prehospital or pre-hospital or out-of-hospital or out of hospital or emergency health service or emergency medical service or EMS or paramedic or emergency medical technician or EMT or prehospital care or emergency medical care or emergency health care or acute medical care or acute care or advanced trauma life support care or ATLS or ATLSC or emergency medicine or first responders or rescue workers or emergency personnel or emergency preparedness).mp. [mp=title, abstract, heading word, table of contents, key concepts, original title, tests & measures, mesh] |
| #16 | #13 AND #14 AND #15 |
| #17 | 16 use psyh* |
| #18 | Use emczd, medall, psyh |
| #19 | #5 OR #12 OR #17 |
| #20 | remove duplicates from #19 |
