# Supplementary material for: Non-medical factors in prehospital resuscitation decision-making: a mixed-methods systematic review
Source: Scand J Trauma Resusc Emerg Med. 2022 Mar 28;30:24. doi: 10.1186/s13049-022-01004-6 (PMC8962561; doi:10.1186/s13049-022-01004-6)
Supplement: Supplementary file 3 — Additional file 3. List of search terms. [file 13049_2022_1004_MOESM3_ESM.docx]

Non-medical factors in prehospital resuscitation decision-making: A systematic review and narrative synthesis

**Search terms**

| **PUBMED** | |
| --- | --- |
| **Category** | **Search words** |
| **Decision-making** | **MeSH**: Decision Making; Clinical Decision-making; Withholding Treatment; Resuscitation Orders  **FRITEKST**  Decision-making; Decisions; Termination; Withhold*; Judgement; Critical thinking; Decision process; Choice behaviour; Ethical decision making; Withdraw; Terminate; Resuscitation orders; Medical futility; Coping behaviour; DNR; Do-not-resuscitate; Advance directive; Living Will; |
| **Cardiac arrest/Resuscitation** | **MeSH**: Cardiopulmonary resuscitation; Resuscitation; Out-of-hospital Cardiac Arrest; Heart Arrest; Cardiac arrest;  **FRITEKST**  Out-of-hospital cardiac arrest; Out-of-hospital heart arrest; cardiac arrest; Out-of-hospital cardiorespiratory arrest; Sudden death; OHCA; Heart arrest; resuscitation; CPR; cardiopulmonary resuscitation; |
| **Out-of-hospital/Prehospital** | **MeSH:** Emergency Medical Services; Emergency treatment; Emergencies; Emergency responders; Emergency medical technicians; Emergency medicine; Ambulances;  **FRITEKST:**  Prehospital OR pre-hospital OR out-of-hospital OR out of hospital OR emergency health service OR emergency medical service OR EMS OR paramedic OR emergency medical technician OR EMT OR prehospital care OR emergency medical care OR emergency health care OR acute medical care OR acute care OR advanced trauma life support care OR ATLS OR ATLSC OR emergency medicine OR first responders OR rescue workers OR emergency personnel OR emergency preparedness |

| **CINAHL** | |
| --- | --- |
| **Category** | **Search words** |
| **Decision-making** | **SUBJECT TERMS**  (MH "Decision Making+") Decision-making; Thinking; Judgement; Problem Solving; Medical futility;  **FRITEKST:**  Decision-making; Decisions; Termination; Withhold*; Judgement; Critical thinking; Decision process; Choice behaviour; Ethical decision making; ~~Decide~~; Withdraw; Terminate; Resuscitation orders; Medical futility; Coping behaviour; DNR; Do-not-resuscitate; Advance directive; Living will; |
| **Cardiac arrest/ resuscitation** | **SUBJECT TERMS** Heart arrest; Resuscitation; Resuscitation Orders;  **FRITEKST**  Out-of-hospital cardiac arrest; Out-of-hospital heart arrest; cardiac arrest; Out-of-hospital cardiorespiratory arrest; Sudden death; OHCA; Heart arrest; resuscitation; CPR; cardiopulmonary resuscitation; |
| **Prehospital/out-of-hospital** | **SUBJECT TERMS** Prehospital Care; Emergency Medical Services; Emergency Care; Emergency Medical Technician Attitudes; Physicians, emergency; Emergency patients; Emergencies; Emergency Treatment; Emergency Medical Technicians; Rapid response; Emergency Medicine;  **FRITEKST**  Prehospital OR pre-hospital OR out-of-hospital OR out of hospital OR emergency health service OR emergency medical service OR EMS OR paramedic OR emergency medical technician OR EMT OR prehospital care OR emergency medical care OR emergency health care OR acute medical care OR acute care OR advanced trauma life support care OR ATLS OR ATLSC OR emergency medicine OR first responders OR rescue workers OR emergency personnel OR emergency preparedness |

| **EMBASE** | |
| --- | --- |
| **Category** | **Search words** |
| **Decision-making** | **SUBJECT HEADING** Decision making; Thinking; Problem Solving; Treatment withdrawal;  **FRITEKST**  Decision-making; Decisions; Termination; Withhold*; Judgement; Critical thinking; Decision process; Choice behaviour; Ethical decision making; Decide; Withdraw; Terminate; Resuscitation orders; Medical futility; Coping behaviour; DNR; Do-not-resuscitate; |
| **Out-of-hospital cardiac arrest/ prehospital resuscitation** | **SUBJECT HEADING** Out-of-hospital cardiac arrest; resuscitation;  **FRITEKST**  Out-of-hospital cardiac arrest; Out-of-hospital heart arrest; Out-of-hospital cardiorespiratory arrest; Sudden death; OHCA; prehospital resuscitation; resuscitation orders; CPR; cardiopulmonary resuscitation; |
| **Prehospital/out-of-hospital** | **SUBJECT HEADING** Emergency Care; Emergency Health Service; Emergency Medicine; Emergency Treatment; Emergency, patient; Emergency, physician; Emergency, nursing;  **FRITEKST**  Prehospital OR pre-hospital OR out-of-hospital OR out of hospital OR emergency health service OR emergency medical service OR EMS OR paramedic OR emergency medical technician OR EMT OR prehospital care OR emergency medical care OR emergency health care OR acute medical care OR acute care OR advanced trauma life support care OR ATLS OR ATLSC OR emergency medicine OR first responders OR rescue workers OR emergency personnel OR emergency preparedness |

| **MEDLINE*** | |
| --- | --- |
| **Category** | **Search words** |
| **Decision-making** | **SUBJECT HEADING** Decision making; Thinking; Judgement; Problem Solving; Medical Futility; Resuscitation Orders;  **FRITEKST**  Decision-making; Decisions; Termination; Withhold*; Judgement; Critical thinking; Decision process; Choice behaviour; Ethical decision making; Decide; Withdraw; Terminate; Resuscitation orders; Medical futility; Coping behaviour; DNR; Do-not-resuscitate; |
| **Cardiac arrest/ Resuscitation** | **SUBJECT HEADING** Out-of-hospital cardiac arrest; Cardiopulmonary resuscitation; Heart arrest; Resuscitation;  **FRITEKST**  Out-of-hospital cardiac arrest; Out-of-hospital heart arrest; Out-of-hospital cardiorespiratory arrest; Sudden death; OHCA; prehospital resuscitation; prehospital; resuscitation orders; CPR |
| **Prehospital/Out-of-hospital** | **SUBJECT HEADING** Emergency Medical Services; Emergency Treatment; Emergency Medical Technicians; Emergency Medicine  **FRITEKST**  Prehospital OR pre-hospital OR out-of-hospital OR out of hospital OR emergency health service OR emergency medical service OR EMS OR paramedic OR emergency medical technician OR EMT OR prehospital care OR emergency medical care OR emergency health care OR acute medical care OR acute care OR advanced trauma life support care OR ATLS OR ATLSC OR emergency medicine OR first responders OR rescue workers OR emergency personnel OR emergency preparedness |

| **PSYCHINFO*** | |
| --- | --- |
| **Category** | **Search words** |
| **Decision-making** | **SUBJECT HEADING** Decision making; Thinking; Judgement; Problem Solving; Coping behaviour; "Clinical Judgment (Not Diagnosis)"  **FRITEKST:**  Decision-making; Decisions; Termination; Withhold*; Judgement; Critical thinking; Decision process; Choice behaviour; Ethical decision making; Decide; Withdraw; Terminate; Resuscitation orders; Medical futility; Coping behaviour; DNR; Do-not-resuscitate; |
| **Cardiac arrest/ Resuscitation** | **SUBJECT HEADING** Heart disorders: CPR;  **FRITEKST**  Out-of-hospital cardiac arrest; Out-of-hospital heart arrest; Out-of-hospital cardiorespiratory arrest; Sudden death; OHCA; prehospital; CPR; cardiopulmonary resuscitation; |
| **Prehospital/Out-of-hospital** | **SUBJECT HEADING** Emergency Services; Emergency Medicine; Emergency Personnel; Emergency Preparedness; Emergency Management;  **FRITEKST**  Prehospital OR pre-hospital OR out-of-hospital OR out of hospital OR emergency health service OR emergency medical service OR EMS OR paramedic OR emergency medical technician OR EMT OR prehospital care OR emergency medical care OR emergency health care OR acute medical care OR acute care OR advanced trauma life support care OR ATLS OR ATLSC OR emergency medicine OR first responders OR rescue workers OR emergency personnel OR emergency preparedness |

| **COCHRANE LIBRARY** | |
| --- | --- |
| **Category** | **Search words** |
| **Decision-making** | **SUBJECT HEADING** Decision making; Resuscitation Orders; Clinical decision making; Withholding treatment; |
| **Cardiac arrest/ Resuscitation** | **SUBJECT HEADING** Out-of-hospital cardiac arrest; Resuscitation; Cardiopulmonary resuscitation; Heart arrest; |
| **Prehospital/Out-of-hospital** | **SUBJECT HEADING** Emergency Medical Services; Emergency Medical Technician; Emergency Responders |
