# Supplementary material for: Non-medical factors in prehospital resuscitation decision-making: a mixed-methods systematic review
Source: Scand J Trauma Resusc Emerg Med. 2022 Mar 28;30:24. doi: 10.1186/s13049-022-01004-6 (PMC8962561; doi:10.1186/s13049-022-01004-6)
Supplement: Supplementary file 4 — Additional file 4. Overview of the synthesis process. [file 13049_2022_1004_MOESM4_ESM.docx]

| **Applying patient related factors and perspectives** | | |
| --- | --- | --- |
| **Subtheme** | **Sub-subtheme** | **Codes** |
| Patient’s characteristics | Patient’s characteristics | More-often initiation and prolonged resuscitation in younger patients (8) |
|  |  | Patients’ age (5, 22, 26, 28, 30, 31, 33, 34, 35, 40, 41, 42) |
|  |  |  |
|  | Social status | Being aware of social status, but not letting it affect decision-making (28) |
|  |  | Social value (30) |
|  | | |
| Ethical aspects concerning the patient | Perceived prognosis | Patient’s QoL (26, 29, 40, 43) |
|  |  | Perceived prognosis (5, 29) |
|  |  | Subjective assessment determined by patient assessment and scene clues (21) |
|  |  | Patient’s appearance (33) |
|  |  |  |
|  | Dignity | Upholding the patient’s dignity (24) |
|  |  | To die with dignity (28, 43) |
|  |  | Patient’s dignity (28) |
|  |  | Life is sacred (28) |
|  |  | To die without interference (28) |
|  |  |  |
|  | Patient’s wishes | Respecting patient wishes (22) |
|  |  | Patient’s wishes expressed by a proxy (41) |
|  |  | Advance directives (21, 27, 32, 34, 35, 38, 40, 41, 42, 44) |
|  |  |  |
|  | Patient’s best interest | Trying to evaluate the patients’ best interests (21) |
|  |  | Giving the patient the benefit of the doubt (22) |
|  |  | Patient’s best interests (29) |

**Supplementary File 4:** Synthesis process of included studies into themes and subthemes (references: qualitative, quantitative, mixed-method)

| **Involving and involvement of bystanders and family members** | | | | | | |
| --- | --- | --- | --- | --- | --- | --- |
| **Subtheme** | | | **Sub-subtheme** | | **Codes** | |
| Family wishes and emotions | | | Family wishes | | Family wishes and expectations (21, 23, 28, 32, 36, 41, 44) | |
|  |  |  |  |  | Involving relatives in decision-making (28, 40) | |
|  |  |  |  |  | Pressure from relatives (29, 31, 45) | |
|  |  |  |  |  | Patient’s and family’s religion (31) | |
|  |  |  |  | |  | |
|  |  |  | Buying time for the family | | Continuing CPR for the family members (23) | |
|  |  |  |  |  | Giving family members time to realize the patient’s death (23) | |
|  |  |  |  |  | Allowing family to say goodbye (5) | |
|  |  |  |  |  | Family members acceptance of death (28) | |
|  |  |  |  |  | Continuation of CPR creates hope (23, 32) | |
|  |  |  |  |  | Unnecessary emotional trauma (44) | |
|  |  |  |  | |  | |
|  |  |  | Coping with the family’s emotions | | Support for the relatives (31) | |
|  |  |  |  |  | Feeling inadequate in meeting family needs (23) | |
|  |  |  |  |  | Transport to avoid facing relatives (28) | |
|  |  |  |  |  | Being uncomfortable with family reactions (45) | |
|  |  |  |  |  | Reactions from family members created uncertainty (23) | |
|  |  |  |  |  | Dealing with family’s emotional response (32) | |
|  |  |  |  |  | Cultural barriers (36) | |
|  |  |  |  | |  | |
|  |  |  | Identifying with the family | | Identifying with the family (23) | |
|  | | | | | | |
| The presence of bystanders | | | Meeting expectations | | CPR for “show” (23, 26, 35) | |
|  |  |  |  |  | Expectations and perceptions of bystanders (8, 28, 31) | |
|  |  |  |  |  |  | |
|  |  |  | Respecting bystander efforts | | Respecting bystanders’ resuscitation attempt (5, 41) | |
|  |  |  |  |  | Acknowledging bystander CPR (28) | |
| **The personal conditions of providers** | | | | | | |
| **Subtheme** | | **Sub-subtheme** | | | | **Codes** |
| Characteristics and experience | | Characteristics | | | | Type of daily work (22, 39) |
|  |  |  |  |  |  | Level of education (22, 33, 37, 41) |
|  |  |  |  |  |  | HCPs gender (30, 33, 35, 41) |
|  |  |  |  |  |  | HCPs age (33, 41, 44) |
|  |  |  |  |  |  | Specific training (33, 38) |
|  |  |  | | | |  |
|  |  | Experience | | | | Decision-making differences in experiences vs. novice HCP (8) |
|  |  |  |  |  |  | Experience and confidence (21, 22) |
|  |  |  |  |  |  | Inexperienced HCP commence or continue resuscitation more often (21) |
|  |  |  |  |  |  | Experience (22, 23, 31, 37, 38, 40, 41, 42, 43, 44) |
|  |  |  |  |  |  | Previous experiences with successful resuscitation (28) |
|  |  |  |  |  |  | Experience from previous cases (5, 28, 31, 44) |
|  |  |  |  |  |  | Pre-existing influences priming expectations of success (22) |
|  |  |  |  |  |  | Lack of experience (29) |
|  |  |  |  |  |  | Inexperience and uncertainty (30) |
|  | | | | | | |
| Emotions and personal values | | Uncertainty | | | | Making sure nothing is missed (21, 45) |
|  |  |  |  |  |  | Prolonged resuscitation when uncertain (21) |
|  |  |  |  |  |  | Requiring verifiable information (8) |
|  |  |  |  |  |  | Unfamiliar situations lead to uncertainty and prolonged decision making (8) |
|  |  |  |  |  |  | Experience and uncertainty (23, 30) |
|  |  |  |  |  |  | Personal beneficence in situations of clinical uncertainty (27) |
|  |  |  |  |  |  | Initiate CPR per default (28) |
|  |  |  | | | |  |
|  |  | Personal values | | | | Individual and interpersonal factors influence application of formal guidelines (22) |
|  |  |  |  |  |  | Personal and emotional values (8) |
|  |  |  |  |  |  | Responsibility (28) |
|  |  |  |  |  |  | HCP’s religion (31, 33, 38) |
|  |  |  |  |  |  | Heroic value (30) |
|  |  |  | | | |  |
|  |  | Fear of consequences | | | | Fear of legal issues or criticism (22, 28, 31, 38, 43) |
|  |  |  |  |  |  | Fear of working outside practice guidelines (27) |
|  |  |  |  |  |  | Official complaint from relatives (26) |
|  | | | | | | |
| Team interactions | | Team interaction | | | | Team agreement (5, 31, 22, 25) |
|  |  |  |  |  |  | Team members’ emotions (5) |
|  |  |  |  |  |  | Getting advice from others (21) |
|  |  |  |  |  |  | Consulting with a superior (22, 26, 29, 31) |
|  |  |  |  |  |  | Crew composition (22) |
| **Being influenced by external conditions** | | | | | | |
|  | **Subtheme** | | | **Codes** | | |
| EMS work environment | EMS system | | | Organizational support (22) | | |
|  |  |  |  | Reputation of the EMS-system (28, 30) | | |
|  |  |  |  | System-related pressure to save lives no matter what (29) | | |
|  |  |  |  | Inappropriate resource utilization (43) | | |
|  |  | | |  | | |
|  | Training purposes | | | Training and teaching purposes (28, 30, 41) | | |
|  |  | | |  | | |
|  | Provider fatigue | | | Provider fatigue at the end of a shift (22) | | |
|  |  | | |  | | |
|  | Crew safety | | | Crew safety (8, 28, 31, 37, 45) | | |
|  |  |  |  | Experience and feeling threatened (45) | | |
|  |  |  |  | Risk of work-related injuries (22) | | |
|  |  | | |  | | |
|  | Area of service | | | Rural vs urban (35) | | |
|  |  | | |  | | |
| Legislation | Formal guidance | | | Guided by the law (24, 38) | | |
|  |  |  |  | Legal uncertainty (22, 32, 34, 38) | | |
|  |  |  |  | Circumstantial factors influence application of formal guidelines (22) | | |
|  | | | | | | |
| The cardiac arrest setting | Location of arrest | | | Location of arrest (36) | | |
|  |  | | | Perceived prognosis according to cardiac arrest setting (21) | | |
|  |  | | |  | | |
|  | The environment | | | Weather conditions (31, 36) | | |
|  |  |  |  | Environmental conditions (8) | | |
|  |  | | |  | | |
|  | Logistics | | | Logistical limitations (8, 31) | | |
| **Navigating conflicts in the area of tension between key factors** | | | | | | |
| **Subtheme** | | | **Sub-subtheme** | | **Codes** | |
| Conflicts with the law and guidelines | | | Legal and guidelines | | Balancing patient’s wishes and legislation (22, 38, 43) | |
|  |  |  |  |  | Conflict between law and personal values (24) | |
|  |  |  |  |  | Balancing patient’s wishes and guidelines (25) | |
|  |  |  |  |  | Grey areas vs. protocol adherence (22) | |
|  |  |  |  |  | Moral decisions vs. protocol (37) | |
|  | | | | | | |
| Conflicting values | | | Family wishes | | Family wishes vs patient rights (22, 24, 25) | |
|  |  |  |  |  | Family wishes vs. resuscitations providers personal values (28) | |
|  |  |  |  |  | Family’s wishes vs patient’s best interest (25) | |
|  |  |  |  |  | Family wishes vs. DNR (31, 32) | |
|  |  |  |  | |  | |
|  |  |  | The duty to save lives | | Conflict between own moral beliefs and system expectations (29) | |
|  |  |  |  |  | Balancing duty and values (23) | |
|  |  |  |  | |  | |
|  |  |  | Team interaction | | Conflicting personal values (in the resuscitation team) (8, 30) | |
|  |  |  |  |  | Conflicting interpersonal factors (22) | |
|  |  |  |  |  | Interdisciplinary conflicts in the team (5) | |
|  |  |  |  | |  | |
|  |  |  | Lack of information | | Well-founded decision versus futile care (5) | |
|  |  |  |  |  | Incomplete or conflicting information (8) | |

**References**

5. Leemeyer AR, Van Lieshout EMM, Bouwens M, Breeman W, Verhofstad MHJ and Van Vledder MG. Decision making in prehospital traumatic cardiac arrest; A qualitative study. *Injury*. 2020;51:1196-1202.

8. Anderson NE, Gott M and Slark J. Grey areas: New Zealand ambulance personnel’s experiences of challenging resuscitation decision-making. *International Emergency Nursing*. 2018;39:62-67.

21. Anderson NE, Gott M and Slark J. Beyond prognostication: ambulance personnel's lived experiences of cardiac arrest decision-making. *Emergency medicine journal : EMJ*. 2018;35:208-213.

22. Brandling J, Kirby K, Black S, Voss S and Benger J. Emergency medical service provider decision-making in out of hospital cardiac arrest: an exploratory study. *BMC Emerg Med*. 2017;17:24-24.

23. Bremer A, Dahlberg K and Sandman L. Balancing between closeness and distance: emergency medical services personnel's experiences of caring for families at out-of-hospital cardiac arrest and sudden death. *Prehospital and disaster medicine*. 2012;27:42-52.

24. Davey P, Lees A and Godbold R. Exploring New Zealand paramedic attitudes towards advance directives: An ethical analysis. *Australasian Journal of Paramedicine*. 2016;13:01-10.

25. Karlsson M, Karlsson N and Hilli Y. Ethical dilemmas during cardiac arrest incidents in the patient's home. *Nursing ethics*. 2019;26:625-637.

26. Larsson R and Engström Å. Swedish ambulance nurses' experiences of nursing patients suffering cardiac arrest. *Int J Nurs Pract*. 2013;19:197-205.

27. Lord B, Récoché K, O'Connor M, Yates P and Service M. Paramedics' perceptions of their role in palliative care: analysis of focus group transcripts. *J Palliat Care*. 2012;28:36-40.

28. Naess AC, Steen E and Steen PA. Ethics in treatment decisions during out-of-hospital resuscitation. *Resuscitation*. 1997;33:245-56.

29. Nordby H and Nøhr Ø. The ethics of resuscitation: how do paramedics experience ethical dilemmas when faced with cancer patients with cardiac arrest? *Prehospital and disaster medicine*. 2012;27:64-70.

30. Nurok M and Henckes N. Between professional values and the social valuation of patients: the fluctuating economy of pre-hospital emergency work. *Soc Sci Med*. 2009;68:504-10.

31. de Graaf C, de Kruif A, Beesems SG and Koster RW. To transport or to terminate resuscitation on-site. What factors influence EMS decisions in patients without ROSC? A mixed-methods study. *Resuscitation*. 2021.

32. Waldrop DP, Waldrop MR, McGinley JM, Crowley CR and Clemency B. Managing Death in the Field: Prehospital End-of-Life Care. *J Pain Symptom Manage*. 2020;60:709-716.e2.

33. Druwe P, Monsieurs KG, Piers R, Gagg J, Nakahara S, Alpert EA, van Schuppen H, Elo G, Truhlar A, Huybrechts SA, Mpotos N, Joly LM, Xanthos T, Roessler M, Paal P, Cocchi MN, BjOrshol C, Paulikova M, Nurmi J, Salmeron PP, Owczuk R, Svavarsdottir H, Deasy C, Cimpoesu D, Ioannides M, Fuenzalida PA, Kurland L, Raffay V, Pachys G, Gadeyne B, Steen J, Vansteelandt S, De Paepe P and Benoit DD. Perception of inappropriate cardiopulmonary resuscitation by clinicians working in emergency departments and ambulance services: The REAPPROPRIATE international, multi-centre, cross sectional survey. *Resuscitation*. 2018;132:112-119.

34. Druwé P, Benoit DD, Monsieurs KG, Gagg J, Nakahara S, Alpert EA, van Schuppen H, Élő G, Huybrechts SA, Mpotos N, Joly LM, Xanthos T, Roessler M, Paal P, Cocchi MN, Bjørshol C, Nurmi J, Salmeron PP, Owczuk R, Svavarsdóttir H, Cimpoesu D, Raffay V, Pachys G, De Paepe P and Piers R. Cardiopulmonary Resuscitation in Adults Over 80: Outcome and the Perception of Appropriateness by Clinicians. *J Am Geriatr Soc*. 2020;68:39-45.

35. Haidar MH, Noureddine S, Osman M, Isma'eel H and El Sayed M. Resuscitation of Out-of-Hospital Cardiac Arrest Victims in Lebanon: The Experience and Views of Prehospital Providers. *J Emerg Trauma Shock*. 2018;11:183-188.

36. Hick JL, Mahoney BD and Lappe M. Factors influencing hospital transport of patients in continuing cardiac arrest. *Annals of emergency medicine*. 1998;32:19-25.

37. Johnson DR and Maggiore WA. Resuscitation decision making by New Mexico emergency medical technicians. *The American journal of emergency medicine*. 1993;11:139-142.

38. Leibold A, Lassen CL, Lindenberg N, Graf BM and Wiese CH. Is Every Life Worth Saving: Does Religion and Religious Beliefs Influence Paramedic's End-of-Life Decision-making? A Prospective Questionnaire-based Investigation. *Indian J Palliat Care*. 2018;24:9-15.

39. Meyer W and Balck F. Resuscitation decision index: a new approach to decision-making in prehospital CPR. *Resuscitation*. 2001;48:255-63.

40. Mohr M, Bahr J, Schmid J, Panzer W and Kettler D. The decision to terminate resuscitative efforts: results of a questionnaire. *Resuscitation*. 1997;34:51-5.

41. Navalpotro-Pascual J, Lopez-Messa J, Fernández-Pérez C and Prieto-González M. Attitudes of healthcare professionals towards cardiopulmonary resuscitation: Results of a survey. *Medicina intensiva*. 2020;44:125-127.

42. Sam S, Pekmezaris R, Nouryan CN, Tan R, Conrardy A, Ward MF, Schwalberg A, Vij B, Silverman H, Guzik HJ, Lesser ML and Wolf-Klein GP. Survey of emergency medical services professionals' experience with advance directives and medical orders for life-sustaining treatment. *J Am Geriatr Soc*. 2011;59:2383-4.

43. Sherbino J, Guru V, Verbeek PR and Morrison LJ. Prehospital emergency medical services' ethical dilemma with do-not-resuscitate orders. *Canadian Journal of Emergency Medicine*. 2000;2:246-251.

44. Stone SC, Abbott J, McClung CD, Colwell CB, Eckstein M and Lowenstein SR. Paramedic knowledge, attitudes, and training in end-of-life care. *Prehospital and disaster medicine*. 2009;24:529-34.

45. Tataris KL, Richards CT, Stein-Spencer L, Ryan S, Lazzara P and Weber JM. EMS provider perceptions on termination of resuscitation in a Large, urban EMS system. *Prehospital emergency care : official journal of the National Association of EMS Physicians and the National Association of State EMS Directors*. 2017;21:610-615.

46. Brandling J, Kirby K, Black S, Voss S and Benger J. Emergency medical service provider decision-making in out of hospital cardiac arrest: an exploratory study. *BMC Emerg Med*. 2017;17:24.
